# Comparison of the movement behaviour of experienced and novice performers during the Cat exercise

**DOI:** 10.1371/journal.pone.0279104

**Published:** 2022-12-22

**Authors:** Ann Hallemans, Emmanuel Jacobs, Jan Gielen, Luc Van Den Dries, Annouk Van Moorsel, Fabien Buisseret, Frédéric Dierick, Nathalie Roussel

**Affiliations:** 1 Department of Physiotherapy and Rehabilitation Sciences (REVAKI/Movant), Faculty of Medicine and Health Sciences, University of Antwerp, Antwerp, Belgium; 2 Department of Sports Medicine and Radiology, University of Antwerp, Antwerp, Belgium; 3 Research Center for Visual Poetics, University of Antwerp, Antwerp, Belgium; 4 Royal Conservatoire, Artesis Plantijn Hogeschool Antwerpen, Antwerp, Belgium; 5 CeREF-Technique, Mons, Belgium; 6 Service de Physique Nucléaire et Subnucléaire, UMONS, Research Institute for Complex Systems, Mons, Belgium; 7 Laboratoire d’Analyse du Mouvement et de la Posture, Centre National de Rééducation Fonctionnelle et de Réadaptation-Rehazenter, Luxembourg, Luxembourg; 8 Faculty of Motor Sciences, UCLouvain, Ottignies-Louvain-la-Neuve, Belgium; 9 Antwerp HeArts, Healthcare for Artists, University of Antwerp and Antwerp University Hospital, Antwerp, Belgium; University of Memphis, UNITED STATES

## Abstract

Two previous studies showed kinematic differences between novice and experienced performers during unchoreographed movements executed in standing position. However, no study explores if these kinematic differences holds during unchoreographed movements executed in quadrupedal position. The aim of this study is to compare the movement behaviour of experienced and novice performers during an exercise wherein they are challenged to use dynamic and largely unchoreographed movement patterns executed in quadrupedal position. The exercise studied was the *Cat* exercise, in which participants were asked to behave like a feline for 10 minutes. An inventory of the chosen movements and the assessment of their average and coefficient of variation of the ground contact temporal parameters, computed by analysing the tri-dimensional whole-body kinematics of 25 performers (n = 13 novices and n = 12 experienced), was compared according to their experience level. No significant difference was found between the groups for the number of chosen movements, and median or coefficient of variation of ground contact temporal parameters, except for a greater foot/ knee swing coefficient of variation in experienced performers. This suggests that biomechanical constraints induced by quadrupedal position “prevent” a different selection of motor strategies by experienced performers, although the latter can be more variable in their movements.

## Introduction

In recent decades, the importance of biomechanical movement analysis of athletes has grown [1–4]. The study of movement control allows us to understand the physical, behavioral and neural components of movement [5]. Two groups that are highly physically active and deserve further attention are dancers [6–8] and performing artists (i.e., artists who combine dance and theater) [9]. The rapid and extreme movements of the lower and upper extremities in the three directions of space, the jumps, twisting and bending movements impose tremendous forces on the spine and the pelvis [10]. Performing artists must therefore combine extreme demands on their bodies while taking aesthetic issues into account.

Most biomechanical studies conducted on dancers have mainly investigated ‘pre-specified’ movement patterns using ballet [11] or landing techniques [12, 13]. Even if variability is an inherent feature in ballet techniques [14], pre-specified choreographed movements performed by dancers are less variable than voluntary, unpredictable or random unchoreographed movements executed by performers in contemporary dance. However, first, an effective communication between the performer and the observer watching is required and linked to kinematic variability of dance movements that modulates the aesthetic appreciation of the observer [15]. Second, a relationship between kinematic variability of dance movements and experience also exists; the expert is thought to have less joint variability than novices [16]. Third, the existence of a model of an optimal amount of human movement variability has been proposed in [17] and could reflect the adaptability of the neuro-musculoskeletal system during a motor task. In this model, a too high or low variability is a signature of a less adaptable system that is therefore more unstable. By combining the recruitment of performers of different experience levels, whole-body biomechanical modelling, tri-dimensional kinematics, and statistical analyses that account for the variability of natural movement patterns, we hypothesized that it is possible to unravel these unchoreographed movements.

To date, there are very few studies that have looked at the kinematic characteristics of these unchoreographed movements. Two studies examined an exercise commonly used by performers: the *Old Man* exercise [18, 19]. In this exercise, performers are asked to build high muscle tension and tremor throughout the body. This exercise is characterized by an inner tremor and an extremely slow and rigid moving pattern and lasts approximately 20 minutes. A biomechanical analysis of experienced performers during this exercise enabled us to trace the effects of experience, gender and progression on kinematics [19]. More, inexperienced performers showed differences in execution of this exercise when compared before and after taking part in seven training sessions [18].

Despite the existence of these two studies that explored an exercise involving less predictable body movements performed in a upright position, there is still a lack of knowledge about the motor behavior of performers during exercises performed in other positions, e.g. the quadrupedal position. From a motor control perspective, in quadrupedal position, coordination between the upper and lower limbs in humans is due to neural connections between the central pattern generators (CPGs) of the cervical and lumbosacral regions [20, 21]. Furthermore, compared to bipedal gait, less flexibility in the spatiotemporal pattern of spinal cord activity and a decrease in sacral compared to lumbar motor neuron activation has been observed during hand-foot crawling [22]. The development of new knowledge in unchoreographed quadrupedal movements executed by performers with different experience-level could make an important contribution to insights into aesthetic appreciation.

This study aims to compare the movement behaviour of performers during an exercise wherein they are challenged to use a dynamic movement pattern realized in a quadrupedal position: the *Cat* exercise. By doing so, insights into movement strategies (chosen movements: crawling, jump, gait), their execution (ground contact temporal parameters) and variability [23] during such exercises are ought to be acquired. In the *Cat* exercise, the performer is asked to transform into a cat, reprocessing of the famous exercise by Jerzy Grotowski (1933–1999) [24].

It has to be noted that both the *Old Man* and the *Cat* exercises are used as exercises during training sessions and as part of performances. Originally, the cat exercise was developed to stretch the muscles [1]. During Fabre’s version of this exercise, the performer is asked to articulate all segments of the body so as to mimic a cat’s movements. The muscle activity of trunk and extremities is task-specific, meaning that a highly specific sequence exists for each movement. It is the coordination between the shoulder girdle and pelvic girdle, which rotate in opposite directions, which creates the image of a moving feline [2]. Fabre created a set of exercises to train his performers [3, 4]. Motor learning principles are also expected to play and important role during these unchoreographed movements. A well known principle in motor control is Bernstein’s degrees of freedom problem where during the initial phases of motor learning the novice performer is hypothesized to have difficulties in controlling the many degrees of freedom of the human body [25]. A strategy of freezing might be used with less coordination between shoulder and pelvic girdle and more en-bloc movements of the trunk. With increased experience, improvements in coordination are expected [26].

It is hypothesised that the kinematics of performers during a quadrupedal exercise with dynamic movement patterns will be influenced by their experience with Fabre’s method of training during a measurement session, as was previously observed in the *Old Man* exercise [19]. More precisely, the number of chosen movements, and contact durations with the ground selected by the performer or their variability could be experience-dependent.

## Materials and methods

### Study design and experimental set-up

As part of a cross-sectional study, participants were observed in a multidisciplinary motion analysis laboratory (M^2^OCEAN, Antwerpen, Belgium) equipped with a three-dimensional motion capture system made of eight Vicon T10 cameras (Vicon® Oxford, UK, 100 fps, 1 megapixel resolution). The study was conducted with the appropriate approvals for the use of human subjects (Belgian registration number: B300201525082). Written informed consent was obtained before onset of the study.

After a five-minute warm-up, participants were asked to perform the *Cat* exercise [27] while recording whole-body kinematics. In the *Cat* exercise, participants are asked to behave like a feline, i.e. to move with extreme precision (mainly on hands and feet) and to be aware of the spatial occupation of their movements [9]. Unlike previous research on movement control in artistic populations [8, 28], no strict guidelines were given regarding movement outcome, creating an artistic autonomy for the performers while executing the exercises. This artistic autonomy served to encourage a voluntary movement pattern without any external restrictions.

The *Cat* exercise took approximately 10 minutes to perform. Each participant performed the exercise once. For each exercise performed, ten trials of 30 seconds each were recorded, separated by a period without recording (during which the exercise was continued). The limitation in recording time is a technical issue related to the available memory in our system. 300 seconds were finally recorded per participant, for an exercise that lasted 600 seconds. The bias in measurement is limited because we are studying unchoreographed movement. Indeed, in a choreographed sequence, we would have missed the same parts in all participants, leading in an incorrect identification of the various movements performed. Here, we rather record 5 minutes of an unchoreographed sequence instead of 10 minutes, which reduces the number of recorded movements but a priori not their relative abundance and kinematics.

### Participants

The population of this study consisted of two groups: experienced (EXP), and novice (NOV) performers. The experienced performers were recruited from Jan Fabre’s acting company, which is known for its physically demanding theatrical performances [19, 27]. From the fifteen performers of this company, twelve performers met the inclusion criteria: having participated in at least one workshop and one production of Jan Fabre (n = 12 for EXP). No exclusion criteria were used.

A group of novice performers (n = 13 for NOV), without any experience regarding Jan Fabre’s work, was recruited as a sample of convenience via the Antwerp Royal Conservatoire. This group consisted of (pre-)professional actors and dancers but lacking experiences with Fabre’s exercises and training method. Participants were contacted by a member of the Conservatoire staff via email and advertisement, without any pre-selection. No exclusion criteria were used. All participants were free of injury at the time of inclusion.

We stress that both our novice and experienced performers are high-level performers. They have to be seen as novice and experienced regarding the *Cat exercise*, which is part of Jan Fabre’s training and performances.

### Data measurement

**Fig 1:**
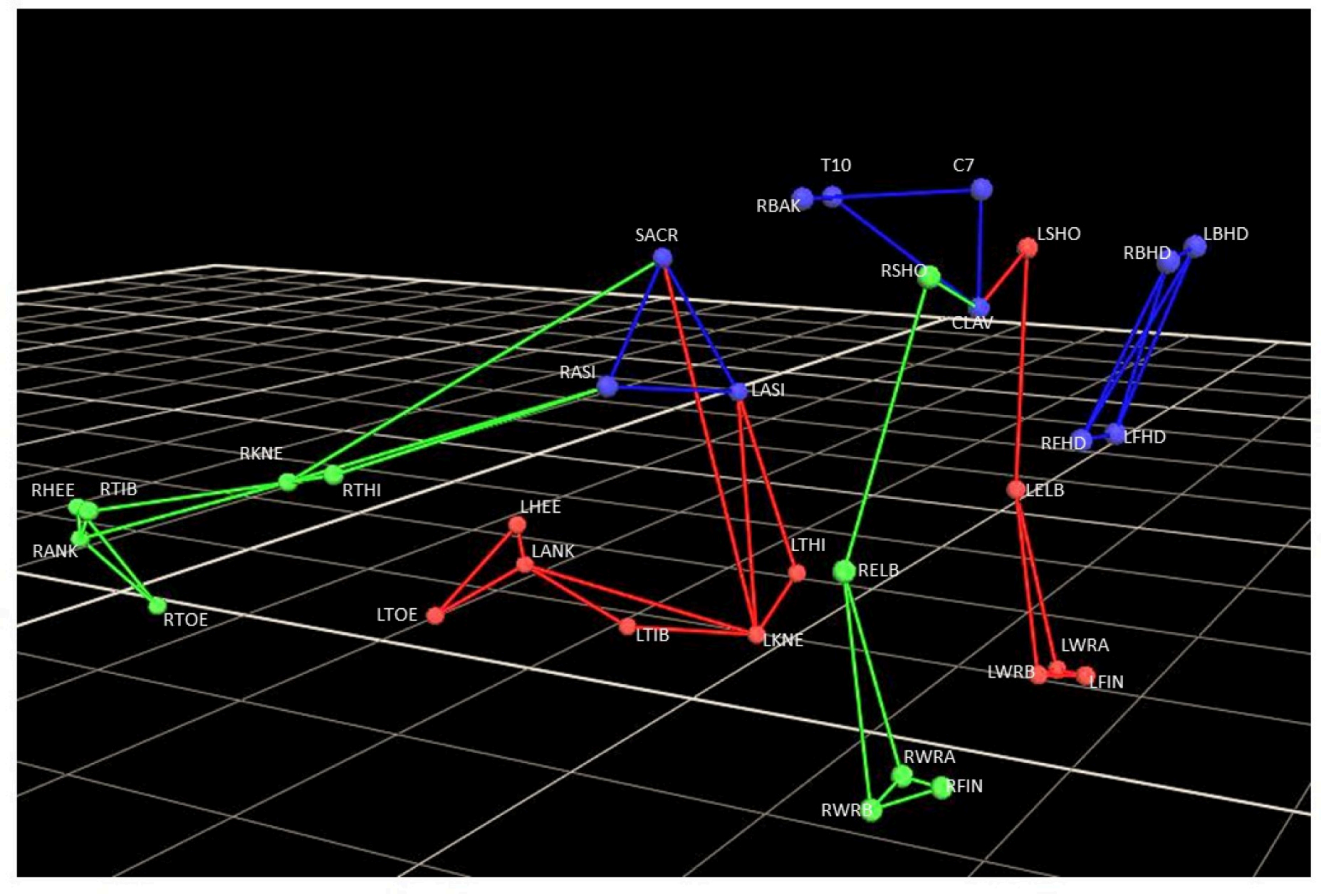
Plug-in Gait marker set-up during *Cat* exercise in novice performer. LFHD = Left front head; RFHD = Right front head; LBHD = Left back head; RBHD = Right back head; C7 = 7th Cervical Vertebrae (spinous process); T10 = 10th Thoracic Vertebrae (spinous process); CLAV = Clavicle (jugular notch); STRN = Sternum (xiphoid process); LASI = Left anterior superior iliac spine; RASI = Right anterior superior iliac spine; SACR = Sacral wand marker. All next markers are placed symmetrically. However, in this legend only the left side markers are named. LSHO = Left shoulder marker (acromio-clavicular joint); LELB = Left elbow (lateral epicondyle); LWRA = Left wrist marker A (styloid process radius); LWRB = Left wrist marker B (styloid process ulna); LFIN = Left fingers (dorsal side of second distal metacarpal head); LTHI = Left thigh; LKNE = Left knee (lateral epicondyle); LTIB = Left tibial wall marker; LANK = Left ankle (lateral malleolus); LHEE = Left heel (calcaneous); LTOE = Left toe (dorsal side of second distal metatarsal head).

Reflective markers were placed over anatomical reference points according to the Plug-In-Gait marker set-up (Fig 1) [29]. Data were captured using Vicon Nexus 1.8 software. After reconstruction and tracking, the marker coordinates were filtered using a second order zero phase-shift low-pass Butterworth filter (cut-off frequency 6 Hz). The conventional gait model [30] was used to obtain 3D kinematic data of the lower and upper extremity joints from the 3D coordinates of each marker.

### Kinematic data analysis

The first step was to determine the foot/ knee and hand cycles based on the analysis of the recorded samples. A foot/ knee (hand) cycle is defined as the time between two consecutive impacts of the left foot/ knee (hand) with the ground. Impact times were determined by visual inspection. Ground contact temporal parameters were then extracted from the dataset. The selected parameters are listed in Table 1. Both magnitude (average values) and variability (coefficients of variation, CV) were calculated from all samples of a given participant, as proposed by [16] for assessing lower limb joint kinematic differences in several dancer groups. CV is computed as standard deviation/average. Obviously, the selected parameters are not exhaustive, but in this first biomechanical study of free quadrupedal movements, we decided to focus on such “standard” kinematical features rather than on more specific parameters depending on time derivatives of the positions such as speed, acceleration, jerk etc.

**Table 1:** Selected hand and foot/ knee ground contact temporal parameters. The left column names the parameter, the second column defines this parameter, and the third column shows how this parameter is expressed. A foot/ knee (hand) cycle is defined as the time between two consecutive impacts of the left foot/ knee (hand) with the ground.

Parameter	Definition	Unit
Foot/ knee support	The time span of contact between foot/ knee and floor	% of foot/ knee cycle
Foot/ knee swing	The time span of non-contact between foot/ knee and floor	% of foot/ knee cycle
Hand support	The time span of contact between hand and floor	% of hand cycle
Hand swing	The time span of non-contact between hand and floor	% of hand cycle
Ipsilateral hand-foot/ knee coordination	The instant during one foot/ knee cycle wherein the same sided hand reaches the floor	% of foot/ knee cycle
Contralateral hand-foot/ knee coordination	The instant during one foot/ knee cycle wherein the opposite sided hand reaches the floor	% of foot/ knee cycle

Based on discussions with Fabre’s team, an inventory was made of the main movements that the performers chose during their execution of the *Cat* exercise. The movements recorded were forward crawl, backward crawl, left turn crawl, right turn crawl, hand-supported jump, full jump, and bipedal gait. Based on this inventory, the chosen movement patterns were counted based on visual inspection. The total number of movements (summed across all trials) was recorded for all the performers.

### Statistical analysis

Statistical analysis was performed in SigmaPlot 13 (Systat Software, San Jose, CA, USA). Trajectories of markers were first explored using boxplots to identify all extreme, irrelevant, values. The latter values were deleted. Missing data (undetected markers) were treated as missing.

A U Mann-Whitney test was used to trace any effects of experience on the medians and CV. For significant p-values at U Mann-Whitney test, effect size was calculated using Cohen’s *d* [31]. Cohen’s *d* values were interpreted as follows [32]: *d* = 0.2 (small), *d* = 0.5 (medium), and *d* = 0.8 (large). Finally, a Chi-squared (χ^2^) test was performed with R free software (v 4.1.0) to compare the total number of movements between the experienced and novice performers. Statistical tests were performed at a significance level of 0.05.

## Results

### Population

The 13 novice performers (1 men, 12 women) had a median Body Mass Index (BMI) of 22.0 kg/m^2^ (minimum = 18.0 kg/m^2^, maximum: 28.0 kg/m^2^). The 12 experienced performers (5 men, 7 women) had a median BMI of 21.5 kg/m^2^ (minimum = 20.1 kg/m^2^, maximum: 24.2 kg/m^2^).

### Experience

Medians and 1^st^ and 3^rd^ quartiles [Q1-Q3] of measured hand or foot/ knee ground contact temporal parameters and their CVs are shown in Table 2 as function of experience (NOV versus EXP). Novice and experienced performers did not differ significantly, except for the foot/ knee swing CV, which was significantly higher in EXP performers (p = 0.047, *d* = 0.86, large effect size).

**Table 2:** Results about hand and foot/ knee ground contact temporal parameters. This table presents the median [Q1-Q3] and coefficients of variation (CV) for temporal parameters of both novice (NOV) and experienced (EXP) groups. The p-values from U Mann-Whitney tests comparing both groups are given. The significant p-value is written in bold font.

Parameter	Group	n		U	p	CV	U	p
Foot/ Knee Support (%)	NOV EXP	13 12	66 [61–72] 67 [61–71]	77	0.978	17 [13–20] 21 [17–26]	46	0.087
Foot/ Knee Swing (%)	NOV EXP	13 12	34 [28–39] 33 [29–39]	77	0.978	35 [25–42] 49 [35–54]	41	**0.047**
Hand Support (%)	NOV EXP	13 12	72 [70–75] 69 [65–74]	54	0.201	12 [8–19] 16 [9–19]	59	0.314
Hand Swing (%)	NOV EXP	13 12	28 [25–30] 31 [26–35]	54	0.201	27 [22–44] 36 [27–41]	70	0.683
Ipsilateral Hand-Foot/ Knee Coordination (%)	NOV EXP	13 12	31 [23–40] 33 [28–37]	71	0.724	43 [28–97] 41 [30–54]	72	0.765
Contralateral Hand-Foot/ Knee Coordination (%)	NOV EXP	13 12	68 [58–76] 71 [66–82]	61	0.369	40 [13–68] 30 [16–47]	67	0.568

### Inventory of chosen movements

**Fig 2:**
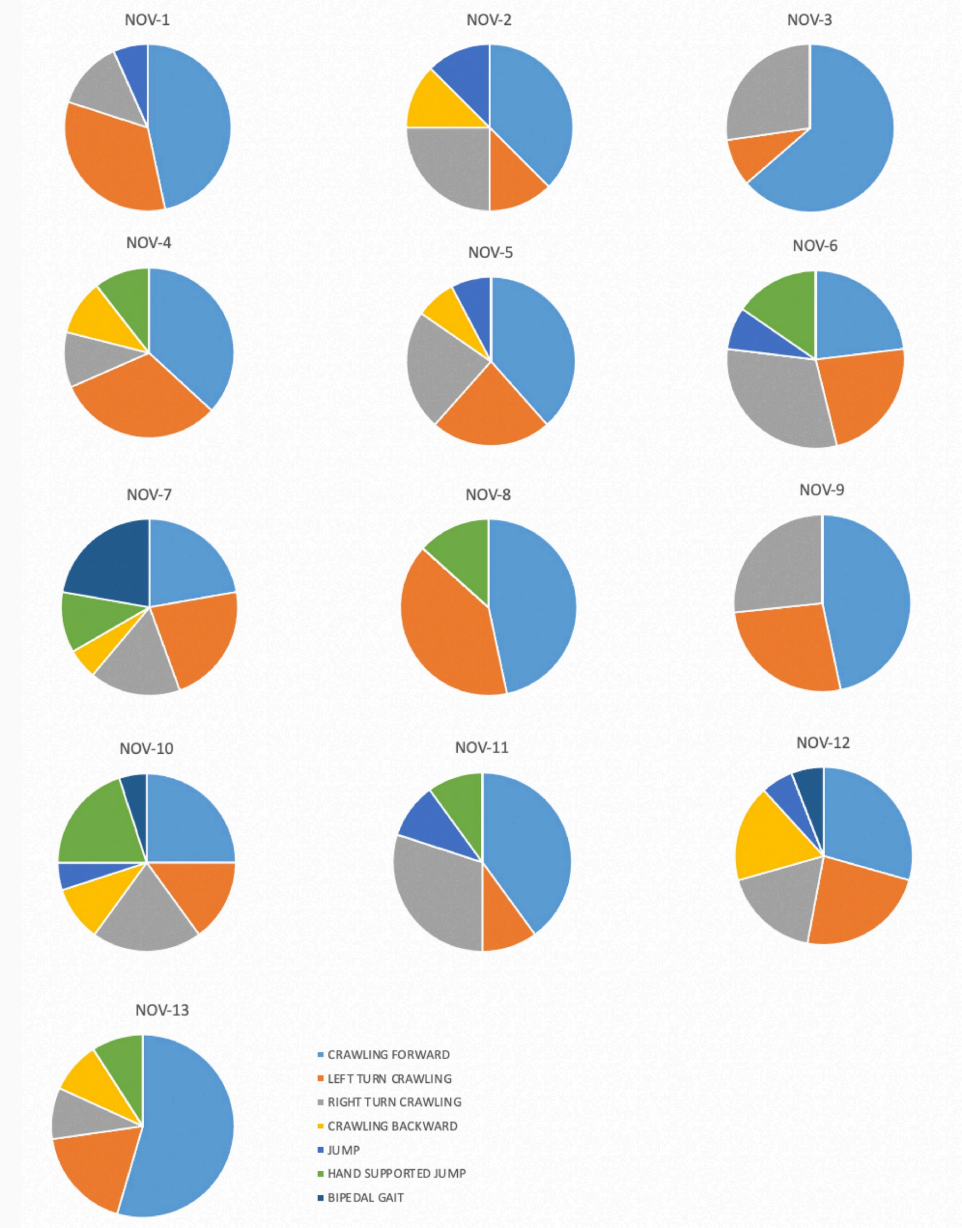
Pie charts for novice performers. These pie charts show the movements chosen by the novice performers: crawling forward, turning left, turning right, crawling backward, jumping, hand-supported jumping, and bipedal gait.

**Fig 3:**
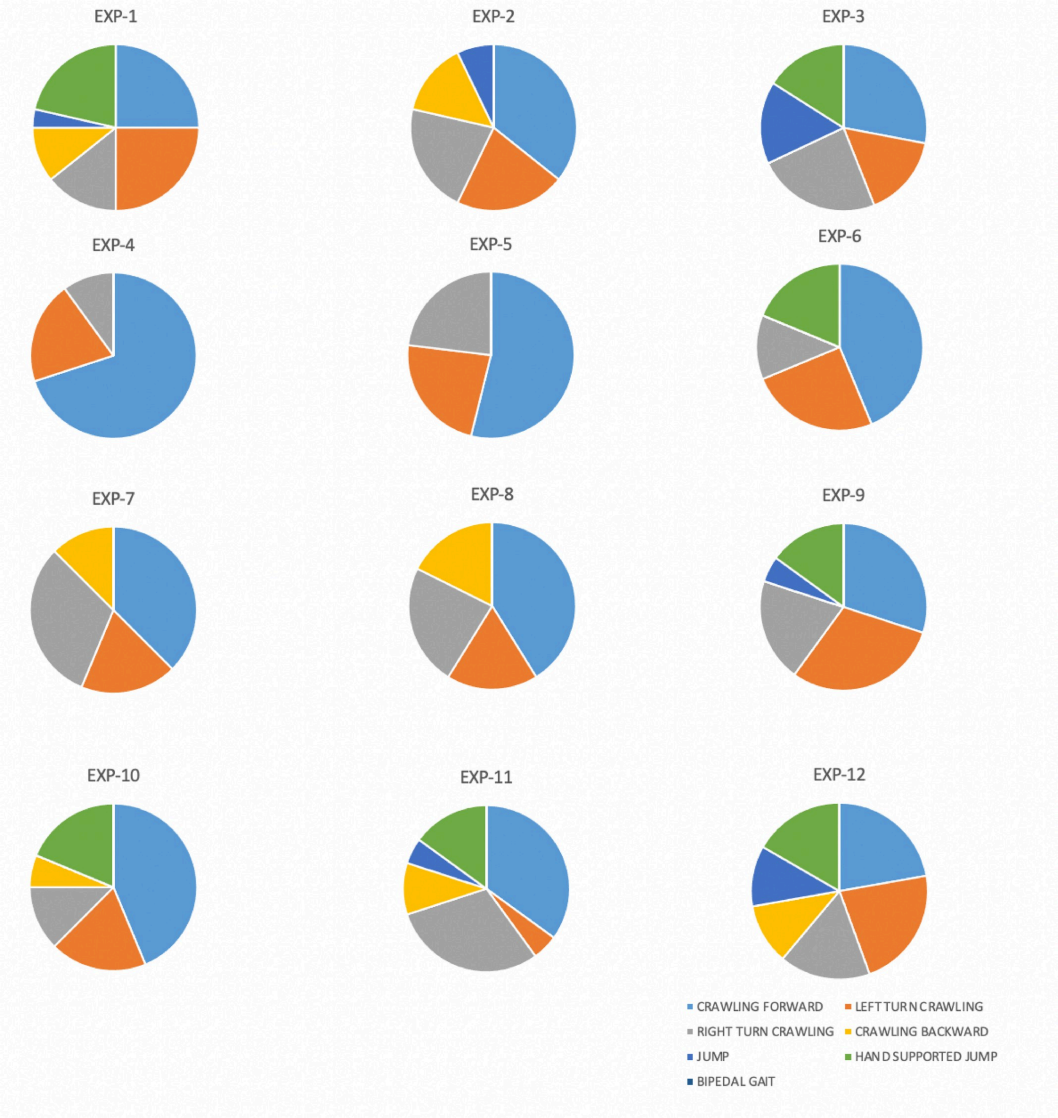
Pie charts for experienced performers. These pie charts show the movements chosen by the experienced performers: crawling forward, turning left, turning right, crawling backward, jumping, hand-supported jumping, and bipedal gait.

Hand-foot/ knee crawling behaviours were observed in performers during *Cat* exercise. Mixed behaviours, with hand-foot on one side and hand-knee on the other were also observed, as jumping or bipedal gait for example. Fig 2 shows pie charts of the movements chosen by the novice performers. Fig 3 shows the same for the experienced performers.

Table 3 shows the type of movements that the performers chose during the exercise. A total of 398 movements were observed with almost a similar number of movements in the two groups. The most chosen movements by the participants were forward and left/ right turn crawling. Note that EXP performers never chose bipedal gait. No significant difference was found in the choice of movement as a function of the performer’s experience (χ^2^ = 9.71, p = 0.137).

**Table 3:** Choice of movements. The first column represents the group within this study’s population: novice (NOV) and experienced (EXP). The next columns represent respectively the number of forward crawling, backward crawling, left turn crawling, right turn crawling, jumping movements, hand-supported jumps and bipedal gait patterns the performers used during the *Cat* exercise. The last column and line show the total values.

	Forward crawling	Backward crawling	Left turn crawling	Right turn crawling	Jump	Hand-supported jump	Bipedal gait	Total (%)
NOV	70	11	43	34	7	14	6	**185 (46)**
EXP	77	15	43	43	10	25	0	**213 (54)**
**Total (%)**	**147 (37)**	**26 (7)**	**86 (22)**	**77 (19)**	**17 (4)**	**39 (10)**	**6 (1)**	**398 (100)**

## Discussion

The aim of this cross-sectional study was to compare EXP and NOV performers during the execution of the *Cat* exercise–regarding which the NOV had no prior experience. The comparison was made at two levels: the choice of movements and the ground contact temporal parameters selected by the performers. Because less flexibility in the spatiotemporal pattern of spinal cord activity has previously been observed in hand-foot/ knee crawling [22], our methodology focused on measuring and analysing the ground contact temporal parameters of the *Cat* exercise, which might differ depending on experience.

Statistical analyses revealed no significant effects of experience on the choice of movements or on median values of ground contact temporal parameters. Yet, the median value of foot/ knee swing CV was 14% higher in EXP performers (p = 0.047) compared to NOV, with a large effect size. It may reflect a better balance of the body in quadrupedal position in experienced dancers, resulting in more variable choices of movements. Opposite variability results for hip external rotation were observed for *Sauté* in first position, with a greater variability in inexperienced dancer groups in this case [16]. This difference may be explained by the highly choreographed component of the *Sauté* that is a ballet figure.

The absence of significant differences in the median ground contact temporal parameters might be explained by the nature of this study’s exercise. Previous studies, examining the *Old Man* exercise, revealed that this exercise is mostly limited to a forwardly directed bipedal gait pattern. Effects of experience and gender are expected in such moving patterns [33–35]. However, the *Cat* exercise leaves more space for other moving patterns than solely forward walking (as seen in Table 3). It also uses a crawling moving pattern, which deviates more from a functional gait pattern. Therefore, it might be less prone to develop a more functional and mature execution with experience. In addition to task related constraints, also biomechanical constraints might play a role. Indeed, it has been shown that ground contact temporal parameters in human quadrupedal gait are highly comparable between crawling infants, children walking on hands and feet, healthy adults and even in pathological quadrupedal gait in case of cerebellar dysfunction (Under Tan syndrome) [36]. In all cases a lateral sequence (ipsilateral hand/foot coordination between 0 and 50%) is preferred, like the timing we observe in EXP and NOV performers but different from the preferred timing in non-human primates. Shapiro and colleagues suggest these preferences should be evaluated in the light of biomechanical constraints, such as ipsilateral limb interference or stability. Regarding variability, it may be said that the significantly higher foot/ knee swing CV in EXP performers suggest that their movement are less stereotyped than those of NOV performers.

As mentioned earlier, one of the possible reasons for the absence of experience-related effects (EXP versus NOV) is likely the large variance within the dataset. It might have been caused by the many different movements that performers chose during their exercise. Therefore, it would be expected that the experience of the performers would not be reflected in any ground contact temporal parameters, but in the choice of movements they performed. However, no differences were found between the EXP and NOV performers based on their movement choices. One of the well-known principles of motor learning, namely the reduction in degrees of freedom, was not recognized in this dataset [37–39] through significant differences in the average ground contact temporal parameters studied.

The results of this study should be seen in the light of some limitations. First, the population consisted of rather small groups. However, considering the exploratory nature of this study, 13 NOV and 12 EXP performers seem to be a reasonable sample size. Since the overall population of performers is also small, such a sample may even be representative of this population. Our findings may more generally apply to performers practicing freely selected movements. Second, only 5 minutes of the *Cat* exercise were recorded and not 10. This may affect the accuracy of the percentages given in Table 3, but we think that a sample 5 minutes already give a satisfactory sample of the total choice of movement due to the short duration of the basic movements identified.

Finally, our *Cat* exercise assessment focuses only on allowed movements, not on emotional state. The influence of performer’s emotions on movement may not be negligible. The influence of emotional factors on the execution of motor tasks in musical performers, athletes, patients and healthy populations is well known from previous literature [40–43]. It is therefore plausible that the performers in the current study experienced the influence of emotional factors, such as fear of failing at the exercise, eagerness to perform the exercise correctly, emotional associations of the exercise with previous experiences, for example. However, objectifying the effects of emotion on movement were not the primary goal of this study. Future research might be valuable to investigate whether other non-physiological (aesthetic/ emotional) factors are helpful in distinguishing between EXP and NOV performers during the *Cat* exercise, for example by using a questionnaire fulfilled by a jury for aesthetic issue or by the performer for emotional state. The assessment of aesthetic issue is now necessary to assess outcome variability [23] of the *Cat* exercise.

## Conclusions

This study expands the knowledge about the importance of biomechanical movement research in the arts. It was able to characterise the *Cat* exercise both in the choice of the selected movements and ground contact temporal parameters. No effect of experience on results was found, excepted in one coefficient of variation which was significantly greater in experienced performers. It appears that the freedom left to the performers in the *Cat* exercise leads to a large variance in the results, making an identification of experience-based strategies not obvious. It may also be that, unlike in the *Old Man* exercise, the quadrupedal position induces biomechanical constraints preventing significantly different voluntary movements in experienced and novice performers.
